# Antimicrobial Activity and Barrier Properties against UV Radiation of Alkaline and Enzymatically Treated Linen Woven Fabrics Coated with Inorganic Hybrid Material

**DOI:** 10.3390/molecules25235701

**Published:** 2020-12-03

**Authors:** Joanna Olczyk, Jadwiga Sójka-Ledakowicz, Anetta Walawska, Anna Antecka, Katarzyna Siwińska-Ciesielczyk, Jakub Zdarta, Teofil Jesionowski

**Affiliations:** 1 The Lukasiewicz Research Network—Textile Research Institute, Brzezinska 5/15, PL-92103 Lodz, Poland; ledakowicz@iw.lodz.pl (J.S.-L.); awalawska@iw.lodz.pl (A.W.); 2 Faculty of Process and Environmental Engineering, Department of Bioprocess Engineering, Lodz University of Technology, Wolczanska 213, PL-90924 Lodz, Poland; anna.antecka@p.lodz.pl; 3 Faculty of Chemical Technology, Institute of Chemical Technology and Engineering, Poznan University of Technology, Berdychowo 4, PL-60965 Poznan, Poland; katarzyna.siwinska-ciesielczyk@put.poznan.pl (K.S.-C.); jakub.zdarta@put.poznan.pl (J.Z.); teofil.jesionowski@put.poznan.pl (T.J.)

**Keywords:** *Cerrena unicolor*, laccase, cellulose fabrics, oxide hybrid, barrier properties, antimicrobial activity

## Abstract

One of the directions of development in the textiles industry is the search for new technologies for producing modern multifunctional products. New solutions are sought to obtain materials that will protect humans against the harmful effects of the environment, including such factors as the activity of microorganisms and UV radiation. Products made of natural cellulose fibers are often used. In the case of this type of material, it is very important to perform appropriate pretreatment before subsequent technological processes. This treatment has the aim of removing impurities from the surface of the fibers, which results in the improvement of sorption properties and adhesion, leading directly to the better penetration of dyes and chemical modifiers into the structure of the materials. In this work, linen fabrics were subjected to a new, innovative treatment being a combination of bio-pretreatment using laccase from *Cerrena unicolor* and modification with CuO-SiO_2_ hybrid oxide microparticles by a dip-coating method. To compare the effect of alkaline or enzymatic pretreatment on the microstructure of the linen woven fabrics, SEM analysis was performed. The new textile products obtained after this combined process exhibit very good antimicrobial activity against *Candida albicans*, significant antibacterial activity against the Gram-negative *Escherichia coli* and the Gram-positive *Staphylococcus aureus*, as well as very good UV protection properties (ultraviolet protection factor (UPF) > 40). These innovative materials can be used especially for clothing or outdoor textiles for which resistance to microorganisms is required, as well as to protect people who are exposed to long-term, harmful effects of UV radiation.

## 1. Introduction

New, ecological solutions rendering textiles bioactive and giving them barrier properties against ultraviolet (UV) radiation are the subject of studies carried out by many research groups [1,2,3,4,5]. An important problem in the refinement and modification of textiles is their appropriate pretreatment before further technological processes.

Use of a biotechnological method of improving the sorption and adhesive properties of textile materials may be an alternative to the previously used processes of pretreatment, such as alkaline treatment or low-temperature plasma. Research is being done into the use of enzymes in finishing processes of textiles made of cellulose fibers [6,7,8,9,10,11]. Flax fibers after retting and scutching contain about 36% non-cellulosic components, including 2% lignin components. The presence of these, due to incrustation on the amorphous part of cellulose, affects fiber rigidity [12,13,14,15]. 

The enzymatic treatment and purification of natural fibers are increasingly important in the preparation of textiles for further finishing processes. This is also reflected in the literature [9], as these methods are environmentally friendly and energy-efficient. Among others, laccase is a very promising enzyme, especially for applications in the textile industry, because it is friendly to the environment. Laccases (EC 1.10.3.2, *p*-diphenolic oxidases) are enzymes that contain multiple copper atoms in their structure. They can be found mostly in fungi, but are also present in smaller amounts in other microorganisms, plants, and even some insects. Laccases were discovered through their ability to oxidize lignin, but they are well known for being able to directly decompose phenols and other aromatic compounds in a way that is harmless to the environment [16,17]. Laccases are widely used in the chemical and environmental industries [18,19], especially for discoloration of wastewater, bleaching processes involving the degradation of lignin, and textile quality improvement through the modification of fiber surfaces or even the synthesis of new dyes [20]. Their broad range of applications and environmentally friendly features mean that laccases are among the most desired naturally obtained enzymes. The results of previous studies have confirmed that laccase from *Cerrena unicolor* was able to degrade most of the examined dyes [18] and was used in a textile bioscouring process [21].

In recent years, there has been increasing research interest in modifiers containing micronized metal oxide particles such as zinc oxide (ZnO), titanium dioxide (TiO_2_) and copper oxide (CuO). Nanoparticles of metal oxides are among the chemical compounds exhibiting bioactivity, absorption of ultraviolet radiation (UV) and the ability to photo-oxidise organic substances [22,23,24]. In addition, TiO_2_-SiO_2_ and ZnO-SiO_2_ oxide hybrids have been increasingly used. The addition of silica can improve the dispersion of metal oxides in the hybrids, reduce the tendency of particles to agglomerate, and contribute to an increase in surface activity. In copper silicate (CuO-SiO_2_), antibacterial and antifungal properties of the copper oxide, as well as viricidal activity, are combined with biocompatibility and nontoxicity. An additional advantage of copper silicate is the possibility of modifying its surface properties using hydrophobic substances and organofunctional compounds [25,26,27,28].

The intense development of nanotechnology enabling the production of micro- and nanostructures has opened up possibilities of producing multifunctional fabrics with a wide range of applications. In recent years, increasing attention has been paid to environmental phenomena and factors having adverse effects on humans. Ultraviolet radiation is perceived as particularly dangerous. One of the ways to eliminate or reduce the harmful effects of UV radiation emitted from natural and artificial sources is the use of textile materials with appropriate barrier properties. These types of materials have the potential to be widely used due to the new properties imparted to the textiles. 

The main aim of this study was to develop textiles made of linen fibers to provide barrier properties against antimicrobial activity and UV radiation, using an innovative treatment that is a combination of bio-pretreatment using laccase from *Cerrena unicolor* and subsequent modification with CuO-SiO_2_ hybrid oxide microparticles. This study of inorganic modifiers should increase understanding of the potential for the application of such modifiers UV barrier in textiles. This is a new phenomenon when compared to the expected and known effects of bioactivity [29]. These innovative materials are able to used especially for clothing or outdoor textiles for which resistance to microorganisms is also required, as well as to protect people who are exposed to long-term, harmful effects of UV radiation.

## 2. Results and Discussion

### 2.1. Enzymatic Activity of Laccase

The proposed methodology of preparation of laccase (see Section 3.2) resulted in an enzyme exhibiting enzymatic volumetric activity of 1–1.5 U/cm^3^ with syringaldazine and 2.5 U/cm^3^ with 2,2′-azino-bis(3-ethylthiazoline-6-sulfonate) acid (ABTS) after 7–10 days of fungal growth. Moreover, filtration and concentration processes resulted in high laccase activity of 10–14 U/cm^3^ with syringaldazine and approximately 26 U/cm^3^ with ABTS. Additionally, samples that were equilibrated to acetate buffer were found to be concentrated more than 10 times, and finally the obtained enzymatic preparation attained the high activity of 17 U/cm^3^ with syringaldazine and 45 U/cm^3^ with ABTS.

The presented improved bioreactor process of *Cerrena unicolor* cultivation proved to be efficient in the production of highly active laccase. The obtained maximal activity of 1.5 U/cm^3^ was higher than in the case of conventional batch fermentation [30]. Additionally, the use of a specially constructed spin filter inside the bioreactor enabled the collection of culture liquid without biomass. As a result, the further purification process was shortened by elimination of the biomass filtration step. 

The present experiments are a continuation of previous work on the bio-scouring of linen fabrics with laccase complex from *Cerrena unicolor* [21]. This time, due to the use of a membrane with a cut-off point of 30 kDa (kilodalton) instead of 10 kDa, a more specific and pure enzymatic product containing highly active laccase was obtained, because a higher number of impurities were separated from the solution. In addition, the further equilibration to acetate buffer made it possible to obtain a concentration about 2.5 times higher than in the previous experiments [21].

### 2.2. Morphological Analysis

It is well known that alkaline treatment is one of the most commonly used methods for cellulose-based textile treatment. The highly alkaline chemicals used for this purpose modify the surface of fibers by removing the impurities such as lignin, hemicellulose, wax, and oils that cover the external surface of natural fibers, but in an oxidative environment also attack the cellulose, leading to a reduction in strength and loss of weight of the fabric. In recent years, there has been intensive research into the use of enzymatic digestion in the technology of chemical processing of textile fibers, as this is a nontoxic, environmentally friendly, sustainable and energy-efficient method [6,7,8,9]. Moreover, the available literature indicates that enzymatic digestion has practical application at various stages of fabric production, including dyeing.

**Figure 1:**
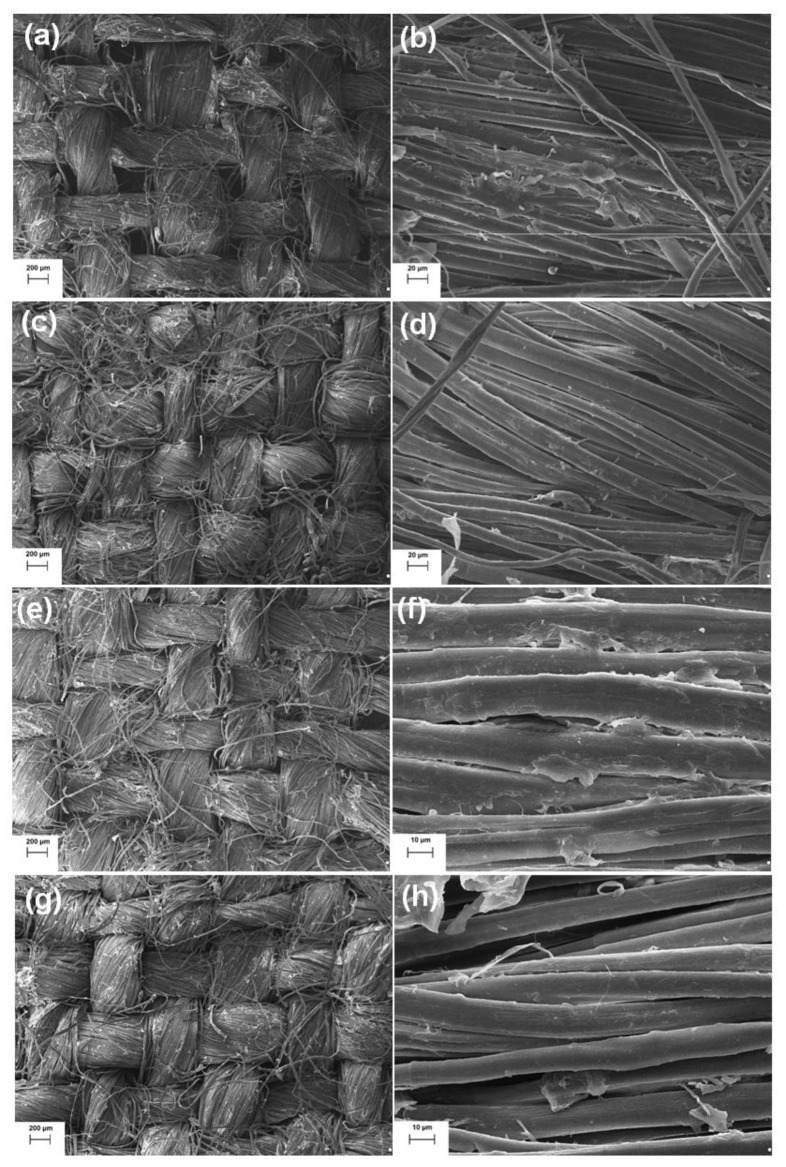
Scanning electron micrographs of: (**a**,**b**) raw linen woven fabric, (**c**,**d**) linen woven fabric after alkaline treatment, (**e**,**f**) linen woven fabric after enzymatic treatment with 2.5 U/g of laccase, (**g**,**h**) linen woven fabric after enzymatic treatment with 5.0 U/g of laccase.

SEM images of raw linen fabric (Figure 1a,b) showed the presence of single fibers with a simple weave, displaying significant similarities. Moreover, the SEM image for untreated linen woven fabric (on Figure 1a) also shows open lumens between single fibers. Morphological analysis showed that the diameter of single fiber is approximately 10 µm. The SEM images of the linen woven fabric after alkaline treatment (Figure 1c,d) showed that this method resulted in polishing (smoothing) of single fibers, which in turn caused their better compaction (on Figure 1c). Morphological analysis showed a single fiber diameter of 10 µm, the same as in the case of raw linen woven fabric. On treatment of linen woven fabrics with laccase from *Cerrena unicolor*, analysis of the morphology of individual fibers revealed significant changes in their structure, which may indicate that the removal of any lignocellulose will be on the ultimate fiber surfaces, and therefore that damage to this layer will produce the observed results. However, it is difficult to determine clearly the degree of their removal (Figure 1e–h). Moreover, SEM images indicated that the treated fabrics were noticeably smoother than the untreated sample. Microscopic observations showed that the laccase removed all protruding surface fibers in comparison with the raw linen woven fabric. It is well known that enzymes cannot penetrate deep inside the fibers, as they have larger sizes than the pores of the fibers. Specifically, enzymes react with their specific substrate on the surface of the sample. These observations coincide with those of Patra and Madhu [12], who report that the enzyme activity is less aggressive and does not cause cellulose degradation. Moreover, those researchers also showed that the results of enzymatic treatment are comparable to those obtained by conventional alkaline etching.

**Figure 2:**
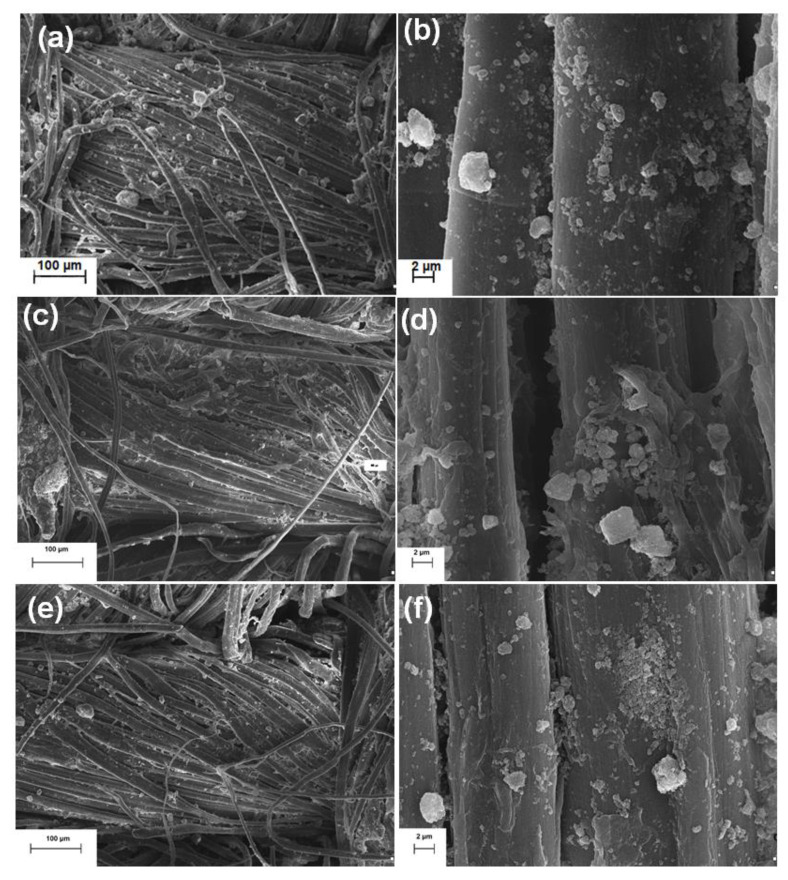
Scanning electron micrographs of: (**a**,**b**) linen woven fabric after alkaline treatment and dip-coating with 5% wt. of CuO-SiO_2_, (**c**,**d**) linen woven fabric after enzymatic treatment with 2.5 U/g of laccase and dip-coating with 5% wt. of CuO-SiO_2_, (**e**,**f**) linen woven fabric after enzymatic treatment with 5.0 U/g of laccase and dip-coating with 5% wt. of CuO-SiO_2_.

In the second stage, to confirm the influence of inorganic modification on the microstructure of alkaline and enzymatically treated linen woven fabrics, scanning electron microscopy was performed (Figure 2). Morphological analysis of the linen woven fabric after enzymatic treatment and dip-coating with CuO-SiO_2_ (Figure 2c–f) showed that the smooth surface of the fibers of the linen fabric is partially covered with dispersed primary particles or agglomerates of the inorganic material, 500–2000 nm in diameter. The SEM images show the presence of both single particles and larger structures’ agglomerates. In addition, in some places, homogeneous coverage of the fiber surface with inorganic material can be observed. 

### 2.3. Barrier Properties of Linen Woven Fabrics against UV Radiation

**Figure 3:**
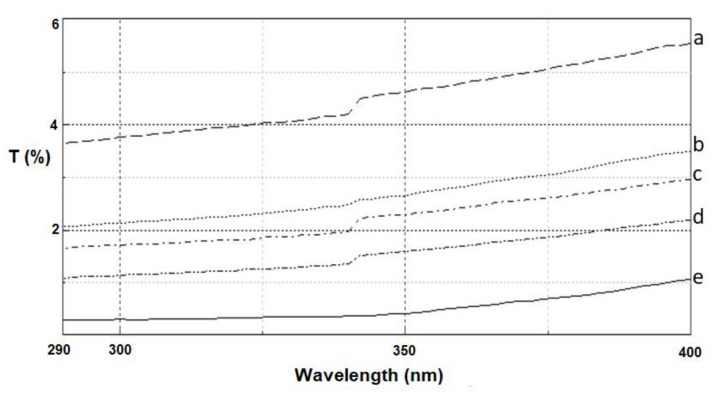
Transmittance spectra (%T) in the range λ = 290–400 nm for: a. raw linen, b. linen woven fabric after dip-coating with 7% wt. of CuO-SiO_2_, c. linen woven fabric after alkaline scouring and dip-coating with 7% wt. of CuO-SiO_2_, d. linen woven fabric after enzymatic treatment with 2.5 U/g of laccase and dip-coating with 7% wt. of CuO-SiO_2_, e. linen woven fabric after enzymatic treatment with 2.5 U/g of laccase and dip-coating with 5% wt. of CuO-SiO_2_.

The results (see Section 3.7) of the protective properties of linen woven fabrics against UV radiation, based on measurement of the ultraviolet transmittance, are shown in Table 1 and Figure 3. 

Table 1 shows the value of the ultraviolet protection factor (UPF) for samples of linen woven fabric following alkaline or enzymatic treatment in addition to modification with CuO-SiO_2_ oxide material. It should be noted that untreated linen woven fabric has an ultraviolet protection factor (UPF) of 27. For this sample, transmittances in the UVA and UVB radiation ranges are 4.52% and 3.9%, respectively. The average transmittance Tav. for linen fabric is 4.22%. Alkaline or enzymatic treatment of linen fabric slightly improves its UV barrier properties. The UPF parameter reached a value of 39 after alkaline scouring and 36 after enzymatic treatment. This can be explained by the reduction in free spaces in the fabric structure, resulting from the more compact structure, as confirmed by the SEM images. Moreover, it was observed that after alkaline or enzymatic treatment of linen woven fabric the average transmittance Tav. decreased to 3.1% or ca. 3.6%, respectively. Incorporation of the inorganic hybrid material on the surface of the linen woven fabric slightly improved its barrier properties. The UPF values are 32 and 29 for linen fabric without pretreatment and modified with 5% or 7% wt. of CuO-SiO_2_, respectively. Moreover, it was observed that a combination of alkaline or enzymatic treatment with inorganic modification of the linen woven fabric (two-step modification) led to improvement of the barrier properties. The UPF parameter reached a value higher than 50. According to the EN 13758-2:2007 standard, textile fabrics have barrier properties against UV radiation if the UPF coefficient is at least 40. The best barrier properties (UPF values of 131 and 128) were obtained for the linen fabrics after enzymatic treatment with 2.5 U/g of laccase and dip-coating with 5% or 7% wt. of CuO-SiO_2_, respectively. In that case, the average transmittance Tav. for fabric after enzymatic (laccase) pre-treatment and dip-coating with 5% or 7% wt. of CuO-SiO_2_ hybrid material takes values in the range 0.31–0.83%. For linen fabrics after alkaline scouring and dip-coating with 5% or 7% wt. of CuO-SiO_2_, poorer values of the UPF parameter were recorded, but these were still 2–3 times higher than for the unmodified fabric. Furthermore, application of CuO-SiO_2_ in the dip-coating process of linen woven fabric after alkaline pre-treatment lowers the average transmittance Tav. to values of 1.52–1.61%.

As previous studies have shown, use of an excessive amount of laccase in the bio-treatment bath decreases the sorption value, and thus the linen fabric possesses worse sorption properties [21]. This may result in a reduction in the amount of modifier adsorbed on the surface of the fibers, which caused a deterioration of the barrier properties against UV radiation: a decrease in the UPF value and an increase in transmittance.

The average transmittance value is a very important parameter which indicates that the modified fabric complies with the criteria for protective/barrier materials against UV radiation (UVA and UVB). The average transmittance (Tav.) in the UVA and UVB range (λ = 290–400 nm) should be lower than 2% for textiles for technical applications and lower than 5% for textiles designed for protective clothing [31]. Figure 3 shows transmittance spectra in the wavelength range λ = 290–400 nm for linen woven fabrics following alkaline or enzymatic treatment or modification with CuO-SiO_2_ hybrid material. The results show that modification of the linen woven fabric with the inorganic hybrid resulted in a significant increase in the absorption of UV radiation in the analyzed range (curve b). Coating a linen fabric with copper silicate particles reduces the transmittance of the sample in the range of UV radiation in comparison with unmodified fabrics. Moreover, it should be noted that after two-step modification of linen woven fabric (combination of enzymatic treatment with 2.5 U/g of laccase and dip-coating with CuO-SiO_2_ in amounts of 5% and 7% wt.; curves e and d) the transmittance in the UVA and UVB range is lower than 2%, which indicates that the obtained fabrics are suitable as textiles for technical applications. It was observed that linen fabric after enzymatic treatment with 2.5 U/g of laccase and dip-coating with 5% wt. of CuO-SiO_2_ exhibits good absorption properties against UV irradiation.

### 2.4. Antimicrobial Activity of Modified Linen Woven Fabrics 

The results of the antimicrobial activity (see Section 3.8) of unmodified and modified linen woven fabrics against selected microorganisms are shown in Table 2. 

The unmodified linen woven fabric shows no antimicrobial activity against any of the examined microorganisms. Fabrics after two-step modification (alkaline or enzymatic treatment and modification with CuO-SiO_2_) display very good antibacterial activity against the Gram-negative bacteria *Escherichia coli*. The strong antibacterial activity of the tested fabrics against *Escherichia coli* was expressed by high values of the antibacterial activity coefficient (A = 4.5–6.3). The microbiological activity coefficient with respect to the Gram-positive bacteria *Staphylococcus aureus* for linen fabrics after alkaline or enzymatic treatment combined with inorganic modification reached values of A = 3–4. Moreover, linen fabrics after two-step modification demonstrated very good antimicrobial activity against *Candida albicans* (antibacterial activity coefficient was higher than 5.3). Furthermore, fabrics after two-step modification (alkaline or enzymatic treatment and modification with CuO-SiO_2_) showed bactericidal activity (value of L coefficient was higher than 0).

The results demonstrate that linen woven fabrics after alkaline or enzymatic pre-treatment and modification with CuO-SiO_2_ in a dip-coating process exhibit increased antimicrobial properties.

## 3. Materials and Methods 

### 3.1. Materials

Raw linen woven fabric (100%, plain weave, mass per unit area 260 g/m^2^, Świat Lnu Sp. z o.o., Kamienna Góra, Poland), sodium silicate (27.18% SiO_2_ and 8.5% Na_2_O, 1390 g/dm^3^ in density, silicate modulus—molar ratio SiO_2_:Na_2_O = 3.3, Vitrosilicon SA, Iłowa, Poland), copper nitrate(V) trihydrate (Cu(NO_3_)_2_*3H_2_O, 99%, Chempur, Piekary Śląskie, Poland), sodium hydroxide (NaOH, 98.8%, Chempur, Piekary Śląskie, Poland), sodium carbonate (Na_2_CO_3_, 99.8%, Chempur, Piekary Śląskie, Poland), sodium chloride (NaCl, 99.9%, Chempur, Piekary Śląskie, Poland), dispersing-sequestering agent Lufibrol^®^ DK (technical, Basf, Ludwigshafen, Germany), sequestering-wetting agent Kieralon CD (technical, Basf, Ludwigshafen, Germany), polyethylene glycol Pluriol^®^ E 400 (technical, average molar mass 400 g/mol, Basf, Ludwigshafen, Germany), hydroxyethylcellulose Cellosize HEC QP-40 (HEC, technical, with low molar mass, DOW, Midland, MI, USA), 2,2′-azino-bis(3-ethylthiazoline-6-sulfonate) acid (ABTS, C_18_H_18_N_4_O_6_S_4_, 98%, Alfa Aesar, Haverhill, MA, USA), syringaldazine (C_18_H_20_N_2_O_6_, 98%, Sigma-Aldrich, Taufkirchen, Germany), acetic acid (C_2_H_4_O_2_, 80%, POCH, Gliwice, Poland). All reagents were used without any further purification. 

### 3.2. Production of Laccase and Its Enzymatic Activity 

The strain of white-rot fungus *Cerrena unicolor* C-139 (culture collection from Department of Biochemistry, Maria Curie-Sklodowska University, Lublin, Poland) was used to produce laccase with high yield without the use of additional inducers. The mycelium grown on plates on 2% malt extract agar (MEA) at 28 °C for 14 days and after that was stored at 4 °C. The production of laccase was performed in a computer-controlled BIOSTAT ED bioreactor (Sartorius, Goettingen, Germany). The total working volume of bioreactor was 15 dm^3^. For cultivation, the Lindeberg–Holm medium with glucose and asparagine was applied according to Janusz et al. [32], thermally sterilized in a bioreactor and inoculated with homogenized mycelium from three overgrown MEA plates with the amount of 240 cm^3^. The process was run for 10 days at a constant temperature of 28 °C with the aeration rate of 2 Ndm^3^ per minute and stirred with the use of a paddle stirrer (Sartorius, Goettingen, Germany) running at 200 rpm. The bioreactor was equipped with a set of sensors (pH and oxygen electrodes, pressure and temperature indicators) to control the process parameters. Additionally, a special rotary filter was installed inside the bioreactor and enabled for sterile collection of fermentation broth containing laccase without the biomass [33]. For biosynthesis, the repeated fed-batch mode was applied with a periodic addition of the substrate and simultaneous collection of the culture media with the enzyme.

The culture broth with crude enzyme was separated from the residual mycelium in a filtration step on paper filters (grade 389, Munktell, Helsinki, Finland). Subsequently, the obtained filtrate with laccase was concentrated and/or purified in an ultrafiltration step using a Vivaflow 50 PES membrane (Sartorius, Goettingen, Germany) with a cut-off point of 30 kDa. The process was continued until the volume of the retentate containing highly active enzyme was reduced tenfold. To increase the efficiency and the stability of enzyme preparation, the samples were equilibrated to 10 mM acetate buffer at pH 5.

Laccase activity was measured spectrophotometrically with the use of a UV/VIS T80+ spectrophotometer (PG Instruments Ltd., Woodway Lane, UK). It was determined in the culture liquid by measuring the oxidation of 300 μM ABTS buffered with 50 mM citrate phosphate (pH 4.5, ɛ_420_ = 36 1/(mM cm); [34]). Additionally, a second substrate was used and activity of laccase was also detected by measuring the oxidation of 0.5 mM syringaldazine dissolved in ethanol buffered with 0.1 M citrate phosphate (pH 5.6, ε_525_ = 65 1/(mM cm); [35]). Activities were expressed in units [U], which represent the formation of 1 μM of product formed per minute under assay conditions and presented as volumetric activity [U/cm^3^].

### 3.3. Synthesis of CuO-SiO_2_ Oxide System

The CuO-SiO_2_ oxide system was fabricated using a precipitation method. The methodology of the synthesis was described in detail by Nowacka et al. [25]. The synthesis of CuO-SiO_2_ material was realized at room temperature applying inorganic sources of silica and cooper oxide as 5% solution of sodium silicate and a 5% solution of copper nitrate, respectively.

### 3.4. Alkaline and Enzymatic Treatment of Linen Fabrics

The linen fabric was subjected to alkaline treatment in an Ugolini laboratory dyeing apparatus at the liquid ratio 10:1, in a bath containing 2 g/dm^3^ of sodium hydroxide, 4 g/dm^3^ of sodium carbonate, 1 g/dm^3^ of dispersing-sequestering agent (Lufibrol^®^ DK) and 1 g/dm^3^ of sequestering-wetting agent (Kieralon CD). The alkaline pretreatment was carried out at a temperature of 98 °C for 60 min. After this stage, the alkali-treated fabric was rinsed for 10 min in a water bath at temperatures of 80, 60 and 40 °C. 

The laccase, which is a multi-copper oxidoreductase enzyme, was used for the enzymatic treatment of linen woven fabrics. This operation was performed in an Ugolini laboratory dyeing apparatus at the liquid ratio 10:1, in a bath containing laccase from *Cerrena unicolor* (with enzyme concentrations of 2.5 and 5.0 U/g of fabric). The enzymatic process was performed at temperature 60 °C and pH 5.3 (phosphate buffer) for 60 min. The process of inactivation of the enzyme was carried out in a water bath at a temperature of 80 °C for 5 min.

### 3.5. Hybrid Inorganic Modification of Linen Woven Fabrics

Because metal oxides and their hybrid materials, unlike dyestuffs, do not show affinity to fibers, they cannot be applied to woven fabrics using typical exhaustion methods. Hence, the incorporation of inorganic particles (nano- and microparticles of copper silicate) into the structure of linen woven fabrics was achieved by a dip-coating method. At the first stage, a water dispersion was prepared containing 5–7% wt. of CuO-SiO_2_ as inorganic material, 10% wt. of polyethylene glycol as a wetting agent and 1% wt. of hydroxyethylcellulose as a thickening agent. The dispersion was vigorously stirred for 60 s using an ULTRA TURRAX T 25 Basic homogenizer, running at a rate of 20,000 rpm (IKA Werke GmbH, Staufen im Breisgau, Germany). Then, the fabric samples were squeezed (using a Benz automatic padding machine) at a nip pressure of 30 kg/cm^2^. At the next step, the woven fabrics were dried and heated at temperature 120 °C for 3 min in a coating-heating machine of type KTF-350 S (Mathis, Oberhasli, Switzerland) [22,23].

### 3.6. Morphological Analysis 

Analysis of the surface microstructure of the fabrics after alkaline or enzymatic pretreatment and additional inorganic modification was performed using an EVO40 scanning electron microscope (Zeiss, Oberkochen, Germany).

### 3.7. Barrier Properties of Linen Woven Fabrics against UV Radiation

Determination of the Ultraviolet Protection Factor (UPF) of textile fabrics after alkaline or enzymatic treatment with the additional incorporation of inorganic particles was performed according to the EN 13758-1 and 2:2007 standards. Barrier properties and the absorption spectra of linen woven fabrics were examined using a Jasco V-550 double-beam type UV-Vis spectrometer (Jasco, Tokyo, Japan) with an integrating sphere attachment.

### 3.8. Antimicrobial Activity of Linen Woven Fabrics 

The antimicrobial activity of woven fabrics was tested against a colony of gram-negative bacteria *Escherichia coli* (American Pure Cultures Collection—ATCC 25922) and gram-positive bacteria *Staphylococcus aureus* (ATCC 6538) and the fungus *Candida albicans* (ATCC 10231) according to American Association of Textile Chemists and Colorists—AATCC Test Method 100-2012—Antibacterial Finish on Textile Materials. This test provides a quantitative procedure for the comparison and evaluation of the degree of antibacterial activity after a 24 h exposure to the tested fabric. After incubation, enumerated and a percent reduction by the fabric specimen is calculated.

Method of calculation of antibacterial activity value according to the standard EN ISO 20743 “Textiles—Determination of antibacterial activity of textile products”:

A = F − G

where: F = lg C_t_ − lg C_0_; F is the growth value on the control specimen (the difference between the lg (logarithm) of the number of grown colonies after the time of contact of the control sample with the inoculum—C_t_, and the lg of the number of grown colonies immediately after applying the inoculum to the control sample—C_0_); G = lg T_t_ − lg T_0_; G is the growth value on the antibacterial testing specimen (the difference between the lg of amount of colonies grown after the time of contacting the sample with antibacterial finish—T_t_, and the lg of amount of colonies grown after application of the sample inoculum finish—T_0_). 

The method of calculation of the bactericidal activity value according to the criteria described in the standard JIS L 1902 “Testing for antibacterial activity and efficacy on textile products” is as follows:

L = lg C_0_ − lg T_t_

L is difference between the lg of the number of grown colonies from the control sample immediately after applying the inoculum to the control sample—C_0_—and the lg of the number of grown colonies from the sample with the finish after the time of contact of the sample with the finish with the inoculum—T_t_.

## 4. Conclusions

The innovative treatment of the woven linen fabric, a combination of bio-pretreatment using laccase from *Cerrena unicolor* and subsequent modification with CuO-SiO_2_ hybrid oxide microparticles by a dip-coating method, produced the anticipated results. 

It has been shown that the proposed methodology of enzymatic preparation of laccase from *Cerrena unicolor* enables the attainment of a high activity value of 45 U/cm^3^.

Enzymatic treatment improved the smoothness of individual fibers. Analysis of the morphology of individual fibers following treatment revealed a violation of their structure, which may indicate the removal of non-cellulosic constituents from the lignin–cellulose fiber matrix of the flax.

In terms of morphology, the effects obtained after the enzymatic treatment are comparable to those obtained after alkaline treatment. Morphological analysis confirmed the presence of single particles and agglomerates of CuO-SiO_2_ on the surface of flax fibers following both enzymatic and alkaline treatment.

Single enzyme treatment with laccase from *Cerrena unicolor*, like the alkaline treatment, led to a relatively small increase in the barrier properties of the linen fabric. This is expressed by an increase in the UPF value and a decrease in transmittance in the UVA and UVB ranges.

Single modification of the linen fabric with the CuO-SiO_2_ oxide hybrid increased the barrier properties to a slightly smaller degree than single alkaline or enzymatic treatment.

None of the applied single processes left the linen fabric fully protected against the harmful effects of UV radiation. Only the combination of two processes—bio-treatment or alkali scouring and application of the CuO-SiO_2_ oxide hybrid—resulted in very good protective properties against UV radiation (UPF ≥ 40, transmittance in the UVA and UVB ranges < 2%). The advantage of bio-treatment over alkaline treatment is its nontoxic nature, lower energy consumption and reduced negative impact on the environment.

It was shown that the presence of CuO-SiO_2_ particles on the surface of flax fibers caused the linen fabric to obtain antimicrobial properties against the Gram-positive bacteria *Staphylococcus aureus*, the Gram-negative *Escherichia coli* and the fungus *Candida albicans*, expressed by strong antibacterial activity.

The newly developed materials can be used to protect people against both solar and artificial UV radiation and the development of microorganisms. They may be used in clothing and in outdoor textile products, such as umbrellas, awnings, and portable covers in the form of light tents and canopies. 

## Figures and Tables

**Table 1:** Values of ultraviolet protection factor (UPF) and transmittance in the UVA (T_UVA_) and UVB (T_UVB_) range for linen woven fabrics after alkaline or enzymatic treatment and modification with inorganic hybrid.

Sample Type	UPF	Transmittance (%)
Raw linen	27	T_UVA_ = 4.52 T_UVB_ = 3.9 Tav. = 4.22
Linen/alkaline scouring	39	T_UVA_ = 3.42 T_UVB_ = 2.53 Tav. = 3.1
Linen/2.5 U/g of laccase	36	T_UVA_ = 3.83 T_UVB_ = 2.89 Tav. = 3.62
Linen/5.0 U/g of laccase	36	T_UVA_ = 3.79 T_UVB_ = 2.87 Tav. = 3.58
Raw linen without pretreatment/5% wt. of CuO-SiO_2_	32	T_UVA_ = 3.94 T_UVB_ = 3.20 Tav. = 3.81
Raw linen without pretreatment/7% wt. of CuO-SiO_2_	29	T_UVA_ = 4.21 T_UVB_ = 3.40 Tav. = 4.03
Linen/alkaline scouring/5% wt. of CuO-SiO_2_	64 (>50)	T_UVA_ = 1.81 T_UVB_ = 1.41 Tav. = 1.61
Linen/alkaline scouring/7% wt. of CuO-SiO_2_	72 (>50)	T_UVA_ = 1.61 T_UVB_ = 1.22 Tav. = 1.52
Linen/2.5 U/g of laccase/5% wt. of CuO-SiO_2_	131 (>50)	T_UVA_ = 0.34 T_UVB_ = 0.20 Tav. = 0.31
Linen/5.0 U/g of laccase/5% wt. of CuO-SiO_2_	110 (>50)	T_UVA_ = 0.41 T_UVB_ = 0.31 Tav. = 0.36
Linen/2.5 U/g of laccase/7% wt. of CuO-SiO_2_	128 (>50)	T_UVA_ = 0.86 T_UVB_ = 0.75 Tav. = 0.83
Linen/5.0 U/g of laccase/7% wt. of CuO-SiO_2_	99 (>50)	T_UVA_ = 0.74 T_UVB_ = 0.69 Tav. = 0.79

**Table 2:** Microbiological activity of linen fabric samples modified by alkaline or enzymatic treatment and dip-coating with CuO-SiO_2_.

Sample Type	Microbiological Activity against *Escherchia coli* (ATCC 11229)		Microbiological Activity against *Staphylococcus aureus* (ATCC 6538)		Microbiological Activity against *Candida albicans* (ATCC 10231)	
	A	L	A	L	A	L
Raw linen	−0.2	−5.0	0.0	−4.1	−1.4	−3.8
Linen/2.5 U/g of laccase/5% wt. of CuO-SiO_2_	4.5	0.9	3.0	0.1	>5.7	>2.0
Linen/2.5 U/g of laccase/7% wt. of CuO-SiO_2_	6.3	1.5	4.0	0.2	5.5	1.8
Linen/alkali-scouring/7% wt. of CuO-SiO_2_	6.3	1.2	4.0	0.5	5.3	1.6

**A**—microbiological activity coefficient, evaluation of antibacterial activity **A** < 0.5—None, 0.5 ≤ **A** < 2 —Weak, 2 ≤ **A** < 3—Significant, **A** ≥ 3—Strong; **L**—bactericidal activity value if L coefficient is not lower than 0 (see Section 3.8).
